# Body mass index and depressive rumination are positively associated with each other only in case of GG genotype of catenin alpha 2 gene rs13412541 variant

**DOI:** 10.1192/j.eurpsy.2022.592

**Published:** 2022-09-01

**Authors:** N. Eszlari, Z. Bagyura, A. Millinghoffer, T. Nagy, G. Juhasz, P. Antal, B. Merkely, G. Bagdy

**Affiliations:** 1 Semmelweis University, Department Of Pharmacodynamics, Budapest, Hungary; 2 Semmelweis University, Heart And Vascular Center, Budapest, Hungary; 3 Budapest University of Technology and Economics, Department Of Measurement And Information Systems, Budapest, Hungary

**Keywords:** perseverative negative thinking, body mass index, catenin alpha 2, depressive rumination

## Abstract

**Introduction:**

Catenin alpha 2 gene (*CTNNA2*) is important in the stability of hippocampal synapses and also in brain development. Our recent paper (Eszlari et al, *Pharmaceuticals* 2021, 14, 850) has demonstrated that rumination on sad mood mediates the association of *CTNNA2* only towards psychiatric symptoms, but not towards cardiovascular risk phenotypes.

**Objectives:**

Our present aim was to test the moderating role of rumination and its two subtypes, brooding and reflection, in genetic associations between *CTNNA2* and the same cardiovascular risk phenotypes.

**Methods:**

633 unrelated subjects from the Budakalasz Health Examination Survey with non-missing phenotypic data, and 160 single-nucleotide *CTNNA2* variants remaining after quality control, were included. Linear regression models were run in Plink 1.9 for separate outcomes of body mass index (BMI), and Framingham risk scores for cardiovascular disease, coronary heart disease, myocardial infarction, and stroke. With each variant, predictors were the variant, rumination or its subtype, the variant x rumination interaction, sex, age, and the top ten principal components of the genome. 100,000 label-swapping max(T) permutation was applied for the interaction term within each analysis.

**Results:**

While no significant interaction term survived the familywise permutation, two trends emerged. Namely, BMI seems to have positive association with rumination and its maladaptive brooding subtype only in case of GG genotype of rs13412541, otherwise no association can be detected.

**Conclusions:**

Although replication is needed in larger samples, the relationship between rumination and BMI, conditional on *CTNNA2* genotype, can be important in atypical depression, thus may contribute to stratification of depressed patients.

**Disclosure:**

The study was supported by the New National Excellence Program of the Ministry for Innovation and Technology from the source of the National Research, Development and Innovation Fund (ÚNKP-21-4-II-SE-1); and by 2019-2.1.7-ERA-NET-2020-00005.

